# Pathogen and host genotype differently affect pathogen fitness through their effects on different life-history stages

**DOI:** 10.1186/1471-2148-12-135

**Published:** 2012-08-02

**Authors:** Emily Bruns, Martin Carson, Georgiana May

**Affiliations:** 1 Department of Ecology Evolution and Behavior, University of Minnesota, Saint Paul, MN, 55108, USA; 2 Graduate Program in Ecology, Evolution and Behavior, University of Minnesota, Saint Paul, MN, 55108, USA; 3 United States Department of Agriculture – Agriculture Research Service, Cereal Disease Laboratory, University of Minnesota, Saint Paul, 55108, USA

**Keywords:** Host-pathogen interactions, Life history, Density-dependence, Fitness, Pathogen reproductive rate, Puccinia coronata, Avena sativa

## Abstract

**Background:**

Adaptation of pathogens to their hosts depends critically on factors affecting pathogen reproductive rate. While pathogen reproduction is the end result of an intricate interaction between host and pathogen, the relative contributions of host and pathogen genotype to variation in pathogen life history within the host are not well understood. Untangling these contributions allows us to identify traits with sufficient genetic variation for selection to act and to identify mechanisms of coevolution between pathogens and their hosts. We investigated the effects of pathogen and host genotype on three life-history components of pathogen fitness; infection efficiency, latent period, and sporulation capacity, in the oat crown rust fungus, *Puccinia coronata* f.sp. *avenae*, as it infects oats (*Avena sativa*).

**Results:**

We show that both pathogen and host genotype significantly affect total spore production but do so through their effects on different life-history stages. Pathogen genotype has the strongest effect on the early stage of infection efficiency, while host genotype most strongly affects the later life-history stages of latent period and sporulation capacity. In addition, host genotype affected the relationship between pathogen density and the later life-history traits of latent period and sporulation capacity. We did not find evidence of pathogen-by-host genotypic (GxG) interactions.

**Conclusion:**

Our results illustrate mechanisms by which variation in host populations will affect the evolution of pathogen life history. Results show that different pathogen life-history stages have the potential to respond differently to selection by host or pathogen genotype and suggest mechanisms of antagonistic coevolution. Pathogen populations may adapt to host genotypes through increased infection efficiency while their plant hosts may adapt by limiting the later stages of pathogen growth and spore production within the host.

## Background

Evolution of differing investment in life-history stages is a powerful mechanism of organismal adaptation to ecological challenges [1], including the challenges imposed on host populations by ever-evolving pathogens [2-4]. While the evolution of host life-history strategies such as earlier maturation and reproductionin response to greater pathogen prevalence has been explored [5-7], the evolution of pathogen life histories in response to host genotypic variationis less well understood. Pathogen life histories have been shown to evolve in response to changes in the abiotic environment [8], shortened host life spans [9], and multiple infections [10]. Although fewer in number, the above studies are important because they demonstrate the potential role of pathogen life history in adaptation to varying ecological factors. What remains unclear is the extent to which varying host genotype might affect the evolution of pathogen life history. Here we examine the effects of host and pathogen genotype on variation in critical stages of a pathogen's life history within the host, spanning infection to reproduction.

Pathogen growth and reproduction within the host are critical parameters in all predictive models because they are strongly correlated with lifetime fitness. While overall reproductive rate is determined by both transmission between hosts and growth and reproduction within the host [11], within-host processes directly affect the evolution of virulence [12-14] and of aggressiveness [11]. Throughout, we use the term virulence to denote the qualitative ability of the pathogen to infect specific host genotypes, and aggressiveness to denote the damage to the host as a result of pathogen infection and growth. Much of the focus of coevolutionary studies has been on qualitative virulence and resistance systems that result in "arms race" evolution [14-18]. For example, the ability to infect is determined by the interaction of pathogen virulence gene products and host resistance proteins [19] in many plant-pathogen systems. Such qualitative systems haveprovided a wealth of information on the classic gene-for-gene coevolutionary processes [15,18,20-23], but the effects of pathogen life -history stages following infection on the coevolutionary process remain understudied. Some authors have hypothesized that qualitative host resistance genes may contribute to quantitative resistance after infection during pathogen growth and reproduction in the host [24,25] and host genotype may not affect all pathogen life-history stages in the same way. For example, Duneau et al. 2011 [26] found that two distinct stages of infection; spore activation and attachment, differed dramatically in host-genotype specificity; activation was not affected by host genotype while spore attachment was strongly affected by the interaction between host and pathogen genotype. For most systems, we have little information on the relative effects of pathogen and host genotype on the quantitative expression of pathogen life-history stages from infection to the production of propagules.

Recent work demonstrates that both pathogen and host genotype affect pathogen reproductive rate in several quantitative systems; murine malaria [27], a protozoan butterfly pathogen [28], an oomycete plant pathogen [29], and two different microsporidium parasites of *Daphnia *[30]. Host and pathogen genotypic interaction effects (GxG interactions) can play a key role in pathogen adaptation to sympatric host genotypes [31-34] as well. Local adaptation is expected in pathogens with dispersal ranges greater than their hosts [18], and there, GxG interactions of pathogen and host genotype may account for a large portion of the variation in pathogen reproductive rate. Explicitly accounting for GxG interactions improves the accuracy of predictive evolutionary models [35-38]. Given the importance of pathogen reproductive rate to disease dynamics in both natural [11,39,40] and agricultural [41,42] systems, untangling the effects of pathogen and host genotype on pathogen life history within the host is an important step towards making accurate predictions of pathogen evolutionary trajectories.

Three pathogen life-history stages are essential to within-host reproduction; infection, the latent period of growth within the host, and finally, propagule production [41]. Together these life-history stages determine a pathogen’s overall reproductive success within the host, however, the contribution of individual life-history stages to overall reproduction can vary among pathogen genotypes [43] and across ecological contexts [8]. Pariaud *et al.*[43] found that the relative contribution of pathogen latent period and sporulation capacity to overall reproductive rate varied among different genotypes of wheat leaf rust. Woodhams *et al.*[8] found that the contribution of specific life-history stages to the overall reproductive rate of the amphibian fungal pathogen *Batrachochyrium dendrobatidis *varied with temperature. At low temperatures, the fungus took longer to mature and developed fewer reproductive structures (zoosporangia) but ultimately produced a large number of zoospores per zoosporangium. At high temperatures, the fungus matured rapidly and developed many more zoosporangia, but produced fewer zoospores per zoosporangium. While there is a growing realization that ecological context will affect the evolution of pathogen life histories [44], and that pathogen life histories strongly affect disease dynamics and coevolution [45], the relative importance of pathogen and host genotype in determining contributions of different life-history stages to pathogen fitness is not well understood.

Here, we investigate the effects of pathogen and host genotype on three life-history stages affecting pathogen reproduction within the host; infection efficiency (the proportion of spores that produce infections), latent period (the lag time between inoculation and spore production that occurs while the fungus grows within the host), and sporulation capacity (the number of spores produced per infection) in the agriculturally important pathogen, *Puccinia coronata* f.sp. *avenae,* the causal agent of oat crown rust. Importantly, to evaluate quantitative genetic effects on within-host pathogen reproduction, we utilized susceptible host genotypes carrying no specific resistance genes preventing infection by the pathogen. We first tested the null hypothesis that only pathogen genotype affects pathogen reproductive rate. Next, we tested the null hypothesis that after infection, qualitative host resistance genes have no effect on pathogen life history and reproductive rate. Finally, we tested the null hypothesis that the relative effects of pathogen and host genotype do not differ across different pathogen life-history stages.

Our results show that pathogen reproductive rate is affected by both the pathogen and the host genotype. We do not find evidence to suggest that a qualitative host resistance gene affects quantitative levels of within-host pathogen reproductive rate. Finally, we show that the relative effects of pathogen and host genotypes vary across pathogen life-history stages with pathogen genotype having the greatest effect in the early life-history stage of infection efficiency, while host genotype has the greatest effect in the later life-history stages of latent period and sporulation capacity.

## Methods

### Study system

In this study, we used the rust fungus, *Puccinia coronata* f.sp. *avenae* (Erik.) as it infects oat (*Avena sativa*) plants and causes crown rust disease. Because the qualitative gene-for-gene interactions are well described for this system [46,47] we could examine the quantitative effects of host and pathogen genotype on pathogen life history and reproduction. Like many rust fungi, *P. coronata* has a complex life cycle in which asexual reproduction occurs on one host (*A. sativa*) and sexual reproduction occurs on a second host (*Rhamnuscathartica*; common buckthorn). Aeciospores produced on the buckthorn host infect oats in early spring and produce pustules on leaves that are filled with bright orange asexual urediniospores, giving the rusts their name. Because urediniospores readily re-infect oats and generation times are rapid (10–14 days), reproductive rate at this spore producing stage is a critical factor in the large epidemics caused by this and many other rust fungi. Consequently, we evaluated the effects of host and pathogen genotype on life-history stages for the urediniospore stage.

We sampled from an experimental nursery for oats and oat crown rust that was established in 1953 in St. Paul, MN and maintained since to test resistance in oat agronomic lines to oat crown rust. The nursery consists of eight 560 m^2^ plots in which over 1,000 different warm and cool season oat lines are planted every year. The oat plots are interspersed with mature hedgerows of buckthorn. Buckthorn plants are inoculated each spring using oat straw from the previous year and the resulting infections on buckthorn produce aeciospores that infect the oats. Thus, we assume that the *P. coronata* population we sampled is representative of a long evolutionary history with oats because of the length of time that the nursery has been in place.

### Pathogen strains

Five different pathogen genotypes (strains) were isolated from collections made in 2008 at the University of Minnesota St. Paul buckthorn nursery. Previous studies have shown that the *P. coronata* population in the nursery harbors high levels of genetic diversity [48,49], and results below]. We collected aeciospores from five different buckthorn plants in the nursery and used standard isolation techniques [49] to insure that each of the fiveresulting strains represents a single genotype. Briefly, aeciospores were used to infect one-week old seedling mixtures of the oat lines Marvelous and Starter because these very susceptible hosts should provide little selection on the pathogen genotype. We used a low inoculum level (1x10^5^ spores/mL) to insure that each resulting infection focus (pustule) producesa single genotype of urediniospores. The leaves of infected oat seedlings were trimmed so that each plant harbored a single pustule. The urediniospores originating from a single pustule represent a single genotype because they are produced by asexual reproduction. The urediniospores produced in each individual pustule were collected and inoculated onto new, uninfected seedlings to produce sufficient quantities of the single-genotype urediniospores for the inoculation experiments described below. These urediniospores were collected, desiccated, and stored at −80°C until used for experiments.

We conducted further evaluations to insure that all five strains were genetically distinct by determining the gene-for-gene virulence alleles carried by each strain. In the gene-for-gene system, when the host plant carries a resistance gene that recognizes a specific pathogen product encoded by the virulence allele, it mounts a resistance response. If the pathogen carries a virulence allele that escapes detection by the host resistance factor, it will successfully infect the host. We tallied the presence or absence of specific virulence alleles in the five pathogen strains by inoculating each strain against a set of 21 oat 'differential' lines that each differ at single resistance gene [48]. We assessed infection 10 days later based on the presence or absence of sporulating pustules.

### Host lines

To examine the effect of host genotype on pathogen reproduction, we chose five susceptible agronomic oat varieties, Ogle, Otana, Pendek38, Pendek, and Portage (Table 1), that had similar heights and times to maturity. All these oat varieties (host lines) have been grown in the buckthorn nursery except Pendek, but many oat lines in the nursery, including Pendek38, carry the Pendek genetic background. Pendek and Pendek38 are reported as isolines that differ only at a single resistance gene, and were included in the experiments to evaluate whether qualitative resistance genes contribute to quantitative host resistance. Pendek38 contains a crown rust resistance gene, *Pc38*, derived from the wild hexaploid oat, *A. sterilis *[50]. We used the highly susceptible oat lines Marvelous and Starter, which are also routinely grown in the buckthorn nursery, to propagate the strains as described above. We did not use them in experimental treatments to avoid confounding effects of inadvertent selection for strains that grew well on these varieties. All seeds were obtained from the USDA Cereal Disease Lab with the exception of the Pendek host line, which was obtained from USDA-ARS National Small Grains Collection in Aberdeen, ID (Table 1). Preliminary inoculation trials confirmed that all host genotypes were susceptible to all pathogen genotypes used in this experiment.

**Table 1:** Oat genotype accessions, year of release, and location

**Host genotype**	**Accession**	**Year released**	**Location developed**
Otana	CIav-1976	1976	Montana/Idaho
Ogle	CIav-9401	1980	Illinois
Pendek	CIav-7801	1960	Netherlands
Pendek38	CN 32995	1968	Manitoba, Canada
Portage	CIav-7107	1960	Wisconsin

Accession numbers are for the USDA Germplasm Resource Information Network (GRIN) except for Pendek38, for which we have listed the accession number for Agriculture and Agri-Food Canada.

### Experimental design

To determine the effects of pathogen and host genotype on pathogen reproduction, each of the five pathogen strains described above was inoculated onto single plants representing each of the five host genotypes. A balanced incomplete split-plot design was used to assign pathogen and host treatments: 50 pots were planted with two different host genotypes per pot and both plants in the pot were inoculated with the same pathogen genotype. Each pathogen genotype was inoculated onto each combination of two hosts. Each pathogen by host genotype combination was replicated four times, for a total of 100 individual plants. Pots were arranged in a completely randomized design in the growth chamber. Thus, pathogen genotype was randomized at the whole-pot level, while host genotype was randomized at the split-pot level. Because of the labor-intensive effort required in making inoculations, half the plants were inoculated on each of two consecutive days, at the same time each day. Two replicates of each host by pathogen treatment were placed in each inoculation day block.

### Experimental procedures

To prepare inoculum for the above experimental design, spores of each pathogen genotype were retrieved from −80°C storage, heat shocked at 40°C for 10 minutes [48] and then inoculated onto a mixture of one-week old susceptible seedlings (Marvelous and Starter). Urediniospores from each strain were collected 13 days later, spores were mixed with mineral oil (Soltrol 170, Philips-Conoco, Houston, TX), and concentrations were estimated from an average of four haemocytometer counts.

To grow plants for the above experimental design, seeds were planted on June 16, 2009 in 14 cm pots filled with pasteurized soil. Two host genotypes were planted 6.5 cm apart per pot. Pots were randomized and placed in a growth cabinet under controlled conditions of 16 h, 22°C day, and 8 h, 18°C night. Pots were arranged in 10 trays, with 5 pots in a tray and watered from the bottom. Three weeks after planting, pots were amended with one tablespoon of ‘Osmocote’ (14 N-14P-14 K) slow-release fertilizer (Scotts Miracle-Gro Co.). An additional supplement of water-soluble 20 N-20P-20 K fertilizer was applied once a week starting 4 weeks after planting.

Plant inoculations were carried out on July 30^th^ and 31^st^, 2009, when plants were beginning to flower. Spores obtained above were diluted to a standard concentration of 1 x 10^5^ spores/mL, an inoculum dose shown to be well below the carrying capacity of the leaf in preliminary experiments. The penultimate leaf of each host was fixed to a board 30 cm from the spray nozzle and covered with a clean paper frame so that only 15 cm of leaf was exposed. Spores were sprayed on to the adaxial leaf surface with a pump sprayer moving at a speed of 7.5 cm/second from the bottom to the tip of the leaf (Scientific apparatus shop, University of Minnesota). A clean spray nozzle was used for each strain. Because not all sprayers deliver exactly the same dose, we estimated the actual inoculation dose for each leaf using a glass microscope slide covered with double-sided sticky tape and fixed to the board such that it was sprayed in line with the leaf. After allowing the inoculum to dry for one hour, plants were placed in a dew chamber with 30 seconds of mist every 2 minutes for 12 hours to stimulate spore germination.

To assess levels of spore viability of each *P. coronata* strain, 500μL of 1x10^6^ spores/mL solution was inoculated onto each of two petri plates with 1.5% water agar (15 g DifcoBacto agar per 1000 mL distilled water), on the same day as plant inoculations. Plates were incubated at room temperature for 12 hours and the numbers of spores that did or did not germinate were counted.

### Life-history measurements

We investigated the effects of pathogen and host genotype on three life-history stages affecting pathogen reproduction within the host: *infection efficiency* (the proportion of inoculated spores that produce sporulating pustules), *latent period* (the lag time between inoculation and spore production), and *sporulation capacity* (the number of spores produced per pustule). The development of pustules was monitored daily beginning seven days after inoculation. From that time, inoculated leaves were examined with a magnifying glass at the same time each day for successive five days and the number of sporulating pustules was recorded.

To estimate total spore production, the inoculated leaf was placed inside horizontally suspended glassine bags adjusted so that spores could not escape, but so that airflow was maintained. No condensation accumulated in the bags during the two weeks that the bags were in place. Spores were collected at 3 weeks after inoculation when pustule development was complete, by tapping the leaves to shake spores into the bags. Any remaining spores were collected from the leaf with a small vacuum and added to those collected in the bag. Spores were desiccated in a 20% relative humidity chamber for one week and weighed. Leaves were harvested, scanned, and total leaf area of the inoculated region was determined using ImageJ [51].

Total spore production was measured as the total dry mass of spores produced and normalized per square centimeter of leaf tissue. Infection efficiency was measured as the number of sporulating pustules per square centimeter of leaf tissue. Latent period was quantified by determining the number of days required for 50 percent of the pustules to reach sporulation. Sporulation capacity was calculated as the average spore mass per pustule. Plant heights from the soil surface to flowering head and from the soil surface to inoculated leaf were measured as potential covariates.

### Statistical analysis

Separate statistical analyses were performed on total spore production and on the three life-history stages contributing to total spore production: infection efficiency, latent period, and spore production per pustule. The effects of pathogen and host genotype on total spore production were determined with a split-plot analysis of variance using inoculation day as a blocking factor. We accounted for variation due to inoculation dose by using the spore counts on the sticky slides (see experimental procedures) as a covariate. The effects of pathogen genotype and of block (inoculation day) were tested using the whole-plot error term, while the effects of host genotype and of inoculum dose were tested using the split-plot error term. We examined all pairwise differences among pathogen and host genotypes using conservative Bonferroni corrections for multiple comparisons, since this method did not change the error structure of the split-plot design. Since two of the host lines, Pendek and Pendek38, were reported as isolines and therefore not truly independent, we ran three analyses; with all five host lines, with the Pendek line excluded, and with the Pendek38 line excluded.

*Infection efficiency* is defined as the proportion of inoculated spores that form sporulating pustules. We measured infection efficiency as the number of pustules per square centimeter of leaf tissue, and accounted for the variation in inoculum dose (spores deposited per cm^2^) using a split-plot ANCOVA model. We used covariate modeling to estimate infection efficiency rather than direct calculation of the ratio of pustules to the inoculum dose because the analysis of covariance explicitly allows a test for density dependent effects. Pathogen genotype effect was tested using the whole-plot error term, while the effects of host genotype, and inoculum dose were tested using the split-plot error term. Results for one leaf were excluded because the sticky slide was missing.

*Latent period*, the time from inoculation until spore production, was calculated by plotting the percentage of sporulating pustules over time, fitting a smoothing line, and graphically determining the time at which 50 percent of the pustules were producing spores (JMP 5.01.a). The percentage of sporulating pustules for each day was calculated by dividing the number of sporulating pustules by the final number of pustules observed 12 days after inoculation (x 100%) when pustule development was complete. Latent period was analyzed with a split-plot ANCOVA, with log transformed pustule density included as a covariate in the model. One leaf was excluded from the analysis since no pustules developed on it.

*Sporulation capacity* is defined as the number of spores produced per pustule [33]. We used a covariate approach to analyze sporulation capacity because our method of collecting spores pooled across all the pustules on a given leaf. We analyzed the log transformed dry spore weight normalized to leaf area (mg of spores per cm^2^ of leaf tissue) in a split-plot ANCOVA with pustule density (pustules per cm^2^ of leaf tissue) as a covariate. Three leaves had fewer than five pustules and were omitted from the analysis as outliers.

We also directly calculated spore production per pustule by dividing the total spore mass by the number of pustules and then determined if spore production per pustule declined with increasing pustule density using regression analysis. Spore production per pustule was transformed to the ¼ power to fit a linear model and pustule density was log transformed. Fourteen replicates produced no measurable spore mass, and were not included in the regression analysis. All analyses were performed in R 2.10.1 (The R Foundation for Statistical Computing 2009) using the aov function and type II sums of squares.

## Results

### Variation in the number of virulence alleles

We found that the five pathogen strains each exhibited a unique infection profile against the 21 differential oat lines tested (see Additional file 1). Individual strains carried between 11 and 16 virulence alleles and, because each carried a unique set of virulence alleles, we conclude that the five strains we used in these experiments represent unique genotypes. We found no significant correlations between the number of virulence alleles carried by a given strain and the quantitative measures of infection efficiency (Slope = 0.0962, t_98_ = 1.636, p = 0.105) or total spore production (slope = 0.0522, t_98_ = 1.693, p =0.0937). There was a slight, but significant, negative correlation between number of virulence alleles and latent period (Slope = −0.06694, standard error = 0.03263, t_97_ = −2.051, p = 0.0429).

### Variation in total spore production

We found significant main effects of both pathogen and host genotype on total spore production, but no significant pathogen by host genotypic interaction (Table 2). Inoculum dose demonstrated a significant positive correlation with total spore production and was used as a covariate in the analysis. Inoculation day (as block) and variation among pots within the growth chamber also had a significant effect on total spore production (Table 2). We did not find significant interactions between inoculum dose and pathogen genotype, or between inoculum dose and host genotype and these interaction terms were dropped from the final model. Excluding one or the other of the Pendek isolines did not change the significance of the results (see Additional file 2).

**Table 2:** Summary of ANCOVA results for total spore production

**Source**	**DF**	**Type II SS**	**F**	**p**
Block	1	3.456	9.714	**0.0032**
Pathogen	4	4.745	3.334	**0.0181**
Whole-plot error	44	15.656	3.198	**0.0014**
Inoculum dose	1	0.460	4.138	**0.0527**
Host	4	3.800	8.539	**0.0002**
Pathogen x Host	16	2.145	1.205	0.3290
Split-plot error	25	2.781		

The response variable is total spore production (mg spores per cm^2^ of leaf tissue) and it was log transformed before analysis. Log transformed inoculum dose (spores deposited per cm^2^) was used as a covariate. The effects of block (inoculation day) and pathogen genotype were analyzed at the whole-plot level, while the effects of inoculum dose, host genotype, and the interaction term, pathogen x host genotype, were analyzed at the split-plot level.

**Figure 1:**
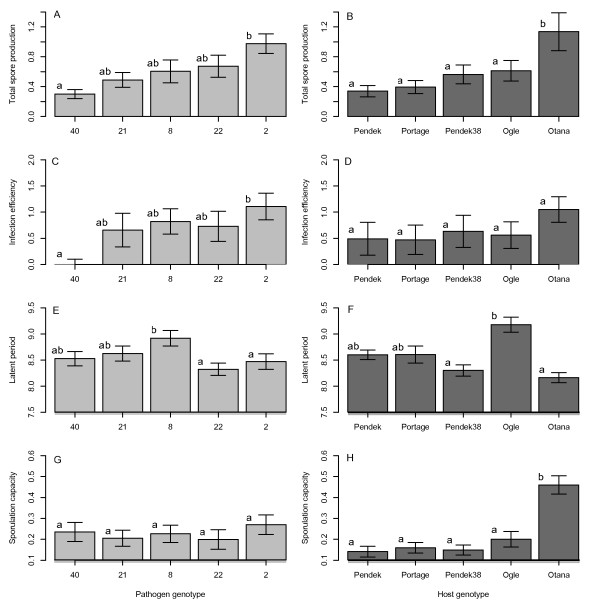
**Main effects of pathogen and host genotype on A, B) total spore production (mg spores per cm**^**2 **^**of leaf tissue), C, D) infection efficiency (log of number of pustules per cm**^**2 **^**of leaf tissue/log of number of spores deposited per cm**^**2**^**), E, F) latent period (days until 50% of pustules reached sporulation), and G, H) sporulation capacity (mg spores per pustule).** Different lower case letters indicate significant differences among mean values (at p = 0.05 level, with Bonferroni correction for multiple comparisons). Error bars represent ± 1 S.E.

Total spore production varied significantly among pathogen genotypes. Pairwise comparisons revealed that strain 2 produced significantly more spores than strain 40 across all hosts (Figure 1A). Spore production also varied significantly among host genotypes (Table 2) with significantly more spores produced on the Otana host genotype than on any other host genotype (Figure 1B). Host genotypes differed significantly in total plant height (F_4,95_ = 12.165, p < 0.0001) and height from the ground to the inoculated leaf (F_4,94_ = 16.017, p < 0.0001) but we did not find a significant correlation between plant height and total spore production or any of the individual life-history stages. Thus, finding that host genotype had a significant impact on total spore production does not support the first null hypothesis that only pathogen genotype affects pathogen reproductive rate, and we turn to evaluating the effects of host and pathogen genotype on separate life-history stages.

### Effect of the host resistance gene Pc38

The Pendek38 host line carries one additional resistance factor, Pc38, compared to the Pendek line. All pathogen strains were able to infect Pendek38, making Pc38 a "defeated" resistance gene (see Additional file 1). We did not detect a significant difference in mean total spore production between the host lines Pendek and Pendek38, nor did we find a significant difference in any of the measured individual pathogen life-history stages between these two host lines. Thus, we accept the second null hypothesis that the qualitative host resistance gene Pc38 has no effect on pathogen life-history stages or on reproductive rate. Since this is a somewhat limited test of the effects of qualitative resistance genes on quantitative pathogen reproduction, we do not pursue this result further.

### Variation in pathogen life-history stages

We evaluated the contributions of three life-history stages to total spore production: infection efficiency, latent period, and sporulation capacity. For each, we asked whether pathogen genotype, host genotype, or the interaction of pathogen and host genotype explained variation in that life-history stage.

#### Infection efficiency

We found that pathogen genotype and inoculation day had significant effects on infection efficiency but that host genotype and the interaction term for pathogen and host genotypic effects were not significant (Table 3). Inoculum dose was a significant predictor of infection efficiency, but the slope of relationship between inoculum dose and infection efficiency did not vary with pathogen genotype. Pathogen strains 2 and 22 produced the most pustules across hosts while strains 21 and 40 produced the fewest across hosts (Figure 1C). When corrected for multiple comparisons, only the difference between strains 2 and 40 was significant.

**Table 3:** Summary of ANCOVA results for infection efficiency

**Source**	**DF**	**Type II SS**	**F**	**p**
Block	1	15.785	131.67	**<0.0001**
Pathogen	4	6.503	13.560	**<0.0001**
Whole-plot error	44	5.275	0.568	0.9504
Inoculum dose	1	1.156	5.478	**0.0275**
Host	14	1.262	0.427	0.9500
Pathogen x Host	16	2.272	0.673	0.7928
Split-plot error	25	5.275		

The response variable is pustule density (pustules per cm^2^ of leaf tissue) and was used as the response variable and it was log transformed before analysis. Log transformed inoculum dose (spores deposited per cm^2^) was used as a covariate. The interaction effect of pathogen genotype by inoculum dose was not significant and was removed from the model.

Differences in infection efficiency among pathogen genotypes could be a result of differences in spore viability at the time of inoculation. To test this, we estimated the germination rate as described above. There was significant variation among pathogen genotypes, despite the fact that all inoculum sources were grown in very similar conditions and that germination rate was above 80% for all genotypes. Surprisingly, strains 21 and 40 had the highest germination rates (97% and 93%, respectively) despite having the lowest infection efficiency. Consequently, spore viability does not explain variation in infection frequency. Inoculation day also had a significant effect, indicating that infection efficiency is sensitive to variation in abiotic environmental conditions.

#### Latent period

We found that both pathogen and host genotype had significant effects on latent period, but that the interaction term for pathogen and host genotypic effects was not significant (Table 4). Pustule density had a significant negative effect on latent period, such that sporulating pustules developed more quickly on more crowded leaves. Inoculation day and pot also had significant effects, indicating that latent period is sensitive to environmental variation (Table 4). Excluding one or the other of the Pendek isolines did not significantly change outcomes of the analysis (see Additional file 3).

**Table 4:** Summary of ANCOVA results for latent period

**Source**	**DF**	**Type II SS**	**F**	**p**
Block	1	8.238	26.612	**<0.0001**
Pathogen	4	3.595	2.903	**0.0323**
Whole-plot error	44	13.621	4.495	**0.0002**
Pustule density	1	2.301	33.407	**<0.0001**
Host	4	5.783	20.993	**<0.0001**
Host x Pustule density	4	0.757	2.746	**0.0556**
Pathogen x Host	16	1.292	1.173	0.3604
Split-plot error	21	1.446		

The response variable is latent period, and it was measured as days until 50% of the pustules were producing spores. Log transformed pustule density (pustules per cm^2^ of leaf tissue) was used as a covariate. The interaction effect of pathogen genotype by pustule density was not significant and was removed from the model.

Latent period varied significantly among pathogen genotypes with strains 2 and 22 having the shortest latent periods on most hosts, and strain 8 taking the longest to develop pustules on most hosts (Figure 1E). Although significant, the difference in latent period among pathogen genotypes was short, with an average of only 15 hours separating the fastest and slowest genotypes under the growth chamber conditions used here. We did not find a significant interaction effect between pathogen genotype and pustule density because all strains demonstrated shorter latent periods on leaves with more pustules (Table 4).

**Figure 2:**
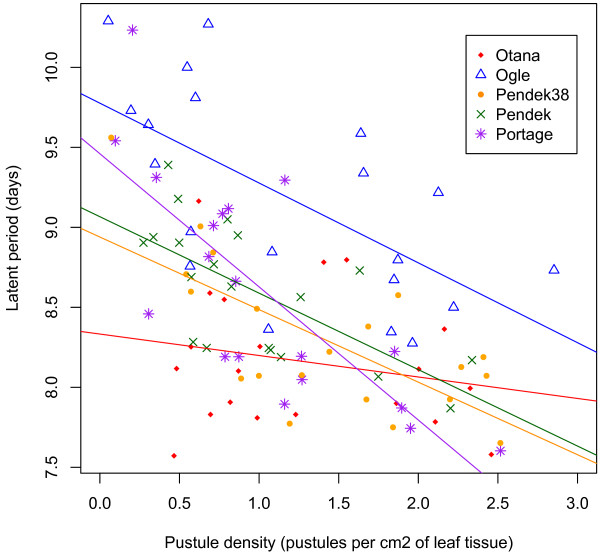
**Effect of host genotype on the relationship between pustule density (pustules per cm**^**2 **^**of leaf tissue) and latent period (days until 50% of the pustules produce spores).** The slope of the regression line for the Portage host genotype is significantly different from the slope of the regression line for the Otana host genotype (t_19_ = −3.017, p = 0.007).

Host genotype had a strong effect on latent period, a result mostly due to the significantly longer latent period of all pathogen genotypes on the Ogle host line (Figure 1F). In addition, latent period for most pathogen genotypes was shorter on the Otana host line compared to the other three hosts, but this was not statistically significant. Interestingly, the longer latent period on the Ogle host line did not correspond to significantly less spore production. Indeed, spore production on the Ogle host line was the second highest. Latent period decreased with increasing pustule density on all host genotypes and there was a marginally significant (p = 0.06) interaction effect between pustule density and host genotype on latent period (Table 4). The slope of the relationship between pustule density and latent period was steepest for the host line Portage (slope = 0.8874, se t_19_ = −3.017, p = 0.006). In contrast, there was no significant relationship between pustule density and latent period on the Otana host (slope = −0.1588, t_19_ = −0.662, p = 0.51) where latent period was consistently short even at lowpustule densities (Figure 2).

#### Sporulation capacity

**Figure 3:**
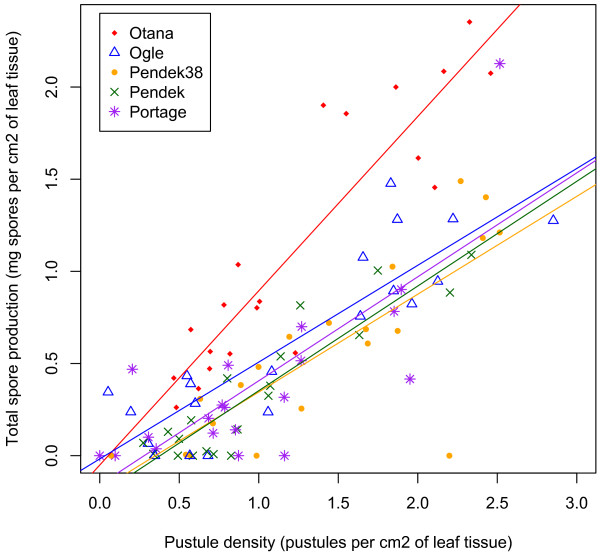
**Effect of host genotype on the relationship between pustule density (pustules per cm**^**2 **^**of leaf tissue) and total spore production (mg spores per cm**^**2**^** of leaf tissue)**. The slope of the regression line for the Otana host genotype is significantly different than the regression slopes for all of the other host genotypes except Pendek (see Additional file 4).

We evaluated sporulation capacity as the relationship between the total mass of spores produced and the number of pustules, normalized to the leaf area (cm^2^). Both pathogen and host genotype had significant effects on sporulation capacity, but the interaction term for pathogen and host genotypes was not significant (Table 5). Pathogen genotype had a significant effect on sporulation capacity but did not affect the slope of the relationship between pustule density and spore production and the interaction term was dropped from the final model. In contrast, host genotype affected both the slope and intercept of the relationship between pustule density and spore production. The slope was steepest on the Otana host genotype (slope = 1.02x10, t_19_ = 2.803, p = 0.012) compared to all other hosts (Figure 3), although no statistical difference in slope was detected between the Otana host genotype and the Pendek host genotype (see Additional file 4). Interestingly, the relationship between spore production and pustule density is strikingly similar among the remaining host genotypes, suggesting a similar underlying physiology in those interactions. Inoculation day and pot also had significant effects (Table 5), indicating that sporulation capacity, like infection efficiency and latent period, is sensitive to environmental variation. Excluding one or the other of the Pendek isolines did not significantly change the outcome of these analyses (see Additional file 5).

**Table 5:** Summary of ANCOVA results for sporulation capacity

**Source**	**DF**	**Type II SS**	**F**	**p**
Block	1	3.616	11.05	**0.0018**
Pathogen	4	4.396	3.36	**0.0175**
Whole-plot error	44	14.404	6.48	**<0.0001**
Pustule density	1	2.189	43.34	**<0.0001**
Host	4	3.438	17.01	**<0.0001**
Host x Pustule density	4	0.618	3.06	**0.0393**
Pathogen x Host	16	0.944	1.17	0.3636
Split-plot error	21	1.061		

The response variable is total spore production (mg spores per cm^2^ of leaf tissue) and it was log transformed before analysis. Log transformed pustule density (pustules per cm^2^of leaf tissue) was used as the covariate. The interaction effect of pathogen genotype x pustule density was not significant and was removed from the model.

We did not see any evidence that spore production leveled off at high pustule densities on any host, indicating that pustule densities were well below carrying capacity of the leaves. When sporulation capacity wasdirectly calculated by dividing the total spore mass by the number of pustules, there was a significant negative correlation between spore mass per pustule and pustule density (slope = −0.0107, t_81_ = −2.801,p = 0.0064) suggesting that crowding might limit sporulation capacity at high densities. One highly influential replicate that only produced a single pustule but had high spore production was removed.

### Pathogen and host genotype effect size

Overall, we found that the amount of variation explained by pathogen and host genotypes varied considerably among the three life-history stages evaluated (Table 6). Pathogen genotype explained more of the variation in infection efficiency (effect size η^2^ x 100% = 24%) than in the later two life-history stages, latent period (14%) and sporulation capacity (17%). Differences in infection efficiency also accounted for most of the among-strain variation in total spore production because pathogen genotypes with the greatest infection efficiency (Figure 1C)produced the greatest total number of spores (Figure 1A). Likewise, pathogen genotypes with the lowest infection efficiency experienced the lowest total spore production (Figure 1A and 1C). In contrast, host genotype had no significant effect on infection efficiency, but explained a large portion of the variation in the later stages of latent period (49.1%) and sporulation capacity (41.7%). Differences in sporulation capacity accounted for most of the among-host variation in total spore production (compare Figure 1B and 1H). Thus, we reject the third null hypothesis that the relative effects of pathogen and host genotype do not differ across different pathogen life-history stages. Pathogen genotype more strongly affected the early stage of infection efficiency while host genotype more strongly affected the later stages of latent period and sporulation capacity.

**Table 6:** **Effect size (η**^**2**^**) of pathogen and host genotype on pathogen life-history stages**

**Life-history stage**	**SS**_**WP**_	**SS**_**P**_	**Pη**^**2**^	**SS**_**SP**_	**SS**_**H**_	**Hη**^**2**^
Total spore production	23.86	4.74	0.199	9.19	3.80	0.414
Infection efficiency	27.56	6.50	0.236	ns	ns	ns
Latent period	25.45	3.59	0.141	11.58	5.78	0.499
Sporulation capacity	22.42	4.40	0.196	8.25	3.44	0.417

Pathogen effect sizes (Pη^2^) were calculated by dividing the sum of squares explained by the pathogen (SS_P_) by the total sum of squares at the whole-plot level (SS_WP_). Host effect sizes (Hη2) were calculated by dividing sum of squares explained by the host (SS_H_) by the total sum of squares at the split-plot level(SS_SP_)_._We did not calculate the effect size for host genotype on infection efficiency because the term was not significant (ns).

## Discussion

Fitness is the currency of natural selection and together with heritable variation, is the basis of phenotypic evolution. Yet measuring fitness is rarely straight forward, particularly for pathogens where fitness cannot be measured outside of the host. In this research, we measuredtotal spore production as an estimate of within-host fitness of the plant pathogen *P. coronata.* Our results are consistent with a growing body of work demonstrating significant effects of both host and pathogen genotype on pathogen fitness [27-30,33,52] and provide new evidence that the effects of host and pathogen genotype vary across the pathogen’s life history within the host.

Host genotype significantly affects total spore production allowing us to reject the null hypothesis that reproductive rate is affected only by pathogen genotype. Instead, our results show that quantitative variation in pathogen reproduction depends on the host genotype and that the magnitude of these host effects varies over the life history of the pathogen in the host. Host genotype had little effect on infection efficiency but strongly affected later life-history stages of latent period and sporulation capacity. Latent periods were significantly longer on the Ogle host line while sporulation capacity was significantly greater on the Otana host line, suggesting differences among these hosts in levels of host defense [53] or resource availability [54,55].

Several life-history stages show apparent density-dependent effects; greater pustule density was correlated with shorter latent periods and lower sporulation capacity. These results are consistent with density-dependent effects observed in other systems where increased pathogen numbers within the host are correlated with lower reproduction per individual [56,57]. Interestingly, we found significant interaction effectsbetween host genotype and density on latent period and sporulation capacity. The interaction effect for latent period arises because pathogen genotypes grown on the Otana host line did not demonstrate decreased latent period with increased pustule density as they did on other host lines. In contrast, the interaction effect for sporulation capacity arises because the relationship of sporulation capacity and pustule density for the Otana host genotype is much steeper than for other host genotypes such that many more spores were produced at high pustule densities. The differences among hosts in density-dependence suggest that the intensity of competition among pustules varies across these host genotypes. We know of no other study demonstrating host genotype effects on the relationship between pathogen density and reproduction. The result is important because competition is thought to select for greater pathogen aggressiveness [58]. If the intensity of pathogen competition varies across a host population, a diverse host population will modulate and slow the evolution of increased pathogen aggressiveness. Further work is needed to determine whether results we show here for single strain infections can be extended to host-mediated effects in multiple strain infections.

Pathogen genotype had significant effects on total spore production and on all three of the life-history stages we measured. The magnitude of these pathogen effects varied over life-history stages, with the strongest effects on variation in infection efficiency and lesser effects on the variation in latent period and sporulation capacity. The differences in infection efficiency among *P. coronata *genotypes could not be easily attributed to spore viability or to a cost of virulence [41,59,60], as the number of virulence alleles carried by a pathogen genotype did not correlate with total spore production. The results do suggest that virulence alleles evolve independently from those affecting quantitative variation in life-history stages. Instead, the differences in infection efficiency among pathogen genotypes may be due to differing abilities to recognize and penetrate the host stomata or to evade basal host defenses [19,53]. Interestingly, although pathogen genotype affects all three life-history stages, we did not observe negative correlations among these traits, thus life-history trade-offs do not obviously limit the evolution of more aggressive genotypes.

Together, the results for host and pathogen genotype show an interesting pattern of decreasing pathogen genotype effects and increasing host genotype effects over the life history of the pathogen. Yet, we found little evidence that pathogen by host genotypic (GxG) interactions affect variation in pathogen life-history stages or total spore production. This result is surprising given the strong main effects of both pathogen and host genotype on latent period and on sporulation capacity. While GxG interactions explain a large portion of the variation in pathogen reproduction in some systems [28,29,33,52], they may have little explanatory value in other systems [27,61]. One explanation for an apparent lack of GxG interactions in our study is that the genetically diverse host populations planted at the sampled location have generated selection for a generalist host-use strategy where most pathogen genotypes infect, grow, and reproduce in most hosts, as has been observed in other studies [62,63]. An alternative explanation is that the small sample of *P. coronata* genotypes and hosts that we used in these experiments lack sufficient variation to detect GxG interactions, although sufficient for detecting main effects. Neither explanation can be excluded but results do suggest that considerable variation in life-history traits has been maintained in this pathogen population.

Variation in pathogen life-history traits is likely maintained through annual sexual recombination [64] and by ecological factors such as the abiotic environment [65], density-dependent effects, and spatial and genetic variation in the host population. Variable abiotic conditions such as occurred over different inoculation days can be a factor maintaining pathogen diversity because environmental effects will limit the effectiveness of directional selection for increased pathogen reproduction rate [65].We observed varied density-dependent effects across host genotypes and conclude that variation in biotic conditions, especially those represented by varied host genotypes, may constrain evolution of life-history traits and maintain variation.

Our results suggest the potential for coevolutionary processes to shape pathogen life-history stages within the host from infection to reproduction. While coevolutionary models have largely focused on qualitative virulence and resistance loci controlling infection [12,14,17,18] we demonstrate that host genotype continues to affect quantitative pathogen fitness following infection. Interestingly, our results indicate that the capacity for pathogen and hosts to respond to selection is different at different life-history stages. Consequently, evolution of increased infection efficiency in the pathogen population might be countered by evolution of host mechanisms limiting sporulation capacity, if lower sporulation increases host fitness. Certainly, different pathogens differ greatly in the mechanisms of reproduction within the host and thus, their detrimental effects on host fitness [39,66,67]. Oat crown rust and other obligate or foliar pathogens that use host resources for their own reproduction [54,68] likely induce selection on the host to reduce their growth and reproduction within the host. In contrast, pathogens that greatly increase juvenile mortality [39,68] or necrotrophs that use toxins to kill the host [69] should induce selection for host mechanisms that reduce the pathogen's infection efficiency or effects of toxins. In any case, examination of the life-history stages most strongly affected by host and pathogen genotype will inform coevolutionary models and improve predictions for the evolution of pathogen aggressiveness.

## Conclusions

Here, we provide evidence for mechanisms by which genetic variation in host populations may drive the evolution of pathogen life-history traits. Complimenting the better-known effects of pathogens on host life histories, our results show that host genotype most strongly affects pathogen life-history stages of growth and reproduction within the host. Moreover, we show that the effects of competition among infections vary with host genotype, suggesting that host population structure has the potential to modulate the evolution of pathogen aggressiveness. We conclude that the capacity of the pathogen or host genotype to respond to selection will be different for different pathogen life-history stages, suggesting an important mechanism of antagonistic coevolution.

## Competing interests

The authors declare that they have no competing interests.

## Authors’ contributions

EB, MLC, and GM were involved in the design of experiments. EB carried out the growth chamber work and statistical analysis. EB and GM drafted the manuscript. All authors read and approved the final manuscript.

## Supplementary Material

Additional file 1 Virulence profile of five pathogen genotypes against 32 differential host lines. Click here for file

Additional file 2 Summary of ANCOVA results for total spore production for datasets with either of the Pendek or Pc38 host genotypes excluded. Click here for file

Additional file 3 Summary of ANCOVA results for latent period for datasets with either of the Pendek or Pendek38 host genotypes excluded. Click here for file

Additional file 4 Pairwise comparison of slopes due to host genotype for the regression of spore production on pustule density. Click here for file

Additional file 5 Summary of ANCOVA results for sporulation capacity for datasets with either of the Pendek or Pendek38 host genotypes excluded. Click here for file
