# Supplementary material for: Pathogen and host genotype differently affect pathogen fitness through their effects on different life-history stages
Source: BMC Evol Biol. 2012 Aug 2;12:135. doi: 10.1186/1471-2148-12-135 (PMC3483255; doi:10.1186/1471-2148-12-135)
Supplement: Additional file 1 — Virulence profile of five pathogen genotypes against 32 differential host lines. [file 1471-2148-12-135-S1.pdf]

Additional file 1. Virulence profile of five pathogen genotypes against 21 differential oat lines.

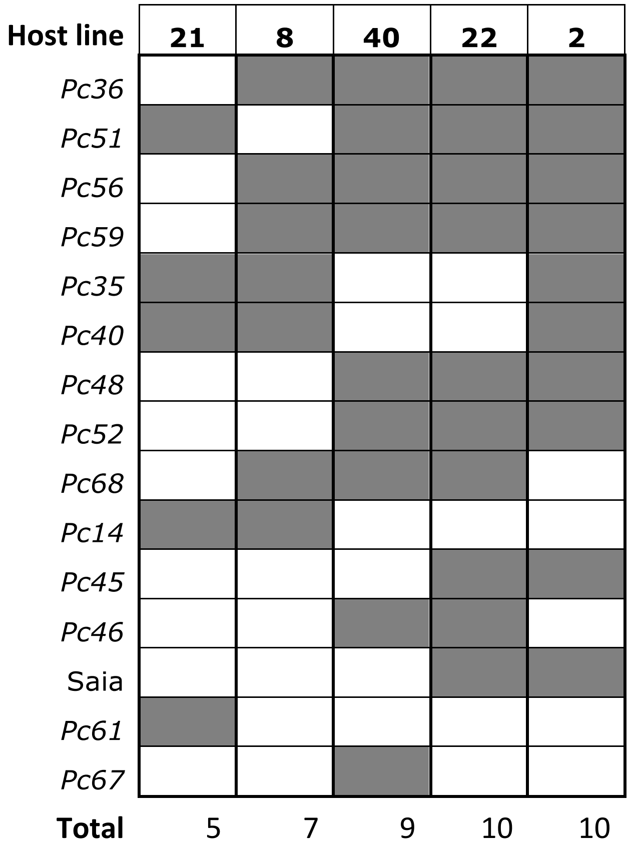

Virulence profile of the five *P. coronata* strains used in the study. Pathogen genotypes are in columns, while oat resistance genes and cultivars are shown in rows. Dark bars indicate successful infection, while white bars indicate a resistant reaction and no infection. The bottom row gives the total number of virulence alleles carried by each pathogen strain, as determined by the number of different host lines infected. 21 host lines were tested but only the 15 host lines that had differential infection of the five strains are shown. All five pathogen strains were virulent on Pc38, Pc39, Pc70, Trisperma, and Bondic, and all five were avirulent on Pc60.
