# Supplementary material for: Pathogen and host genotype differently affect pathogen fitness through their effects on different life-history stages
Source: BMC Evol Biol. 2012 Aug 2;12:135. doi: 10.1186/1471-2148-12-135 (PMC3483255; doi:10.1186/1471-2148-12-135)
Supplement: Additional file 2 — Summary of ANCOVA results for total spore production for datasets with either of the Pendek or Pc38 host genotypes excluded. [file 1471-2148-12-135-S2.pdf]

Additional file 2. Summary of ANCOVA results for total spore production for datasets with either of the Pendek or Pc38 host genotypes excluded.

	Pendek38 excluded				Pendek excluded			
**Source**	**DF**	**Type II SS**	**F**		**DF**	**Type II SS**	**F**	
Block	1	2.747	7.733	***	1	2.882	7.890	***
Pathogen	4	3.965	2.790	*	4	4.026	2.755	*
Whole plot error	44	15.630	2.664	*	44	15.343	4.951	*
Inoculum dose	1	0.538	4.032		1	0.304	4.120	
Host	3	2.700	6.748	***	3	2.695	12.173	***
Pathogen * Host	12	1.332	0.832		12	2.676	3.022	*
Split plot error	12	1.600			13	0.959		

Both the response variable, total spore production (mg per cm^2 of leaf tissue) and the covariate, inoculum dose (spores deposited per cm^2), were log transformed. Asterisks indicate significance at $p < 0.05$, 0.01, and 0.001, respectively. Significance of model effects do not differ from the full model with all 5 host lines included (Table 3).
