# Supplementary material for: Pathogen and host genotype differently affect pathogen fitness through their effects on different life-history stages
Source: BMC Evol Biol. 2012 Aug 2;12:135. doi: 10.1186/1471-2148-12-135 (PMC3483255; doi:10.1186/1471-2148-12-135)
Supplement: Additional file 3 — Summary of ANCOVA results for latent period for datasets with either of the Pendek or Pendek38 host genotypes excluded. [file 1471-2148-12-135-S3.pdf]

Additional file 3. Summary of ANCOVA results for latent period for datasets with either of the Pendek or Pendek38 host genotypes excluded.

	Pendek 38 excluded				Pendek excluded			
**Source**	**DF**	**Type II SS**	**F**		**DF**	**Type II SS**	**F**	
Block	1	6.7669	23.46	***	1	6.8069	19.28	***
Pathogen	4	4.0439	3.51	*	4	3.2353	2.29	
Whole plot error	44	12.6894	4.02	*	44	15.5334	3.69	*
Pustule density	1	0.1164	1.62		1	0.9878	10.31	**
Host	3	7.2406	33.68	***	3	5.0688	17.64	***
Host * density	3	0.7613	3.54	*	3	1.1238	3.91	*
Pathogen * Host	12	0.9853	1.15		12	1.2898	1.12	
Split plot error	10	0.7166			10	0.9577		

Latent period was measured as days until 50% of the pustules were sporulating and log transformed pustule density (pustules per cm^2 of leaf tissue) was used as a covariate. Asterisks indicate significance at $p < 0.05$, 0.01, and 0.001, respectively. Significance of pathogen and host effects do not change from the full model with all 5 cultivars (Table 5). The effect of pathogen genotype is not significant (p=0.075) when Pendek is excluded from the model.
