# Supplementary material for: Pathogen and host genotype differently affect pathogen fitness through their effects on different life-history stages
Source: BMC Evol Biol. 2012 Aug 2;12:135. doi: 10.1186/1471-2148-12-135 (PMC3483255; doi:10.1186/1471-2148-12-135)
Supplement: Additional file 4 — Pairwise comparison of slopes due to host genotype for the regression of spore production on pustule density. [file 1471-2148-12-135-S4.pdf]

Additional File 4. Pairwise comparison of slopes due to host genotype for the regression of spore production on pustule density

**Pairwise comparison**	**Slope**	**Standard error**	**t**	**p**
Otana[a]	0.644	0.230	2.803	**0.012**
Otana vs Ogle[b]	-0.587	0.174	-3.373	**0.004**
Otana vs Pendek38	-0.437	0.186	-2.358	**0.031**
Otana vs Pendek	-0.152	0.177	-0.859	0.403
Otanta vs Portage	-0.413	0.165	-2.510	**0.022**

[a] Estimated slope of the regression between pustule density (pustules per cm^2 of leaf tissue) and total spore production (mg spores per cm^2 of leaf tissue) for the Otana host line.

[b] Difference and standard error of the difference between the estimated slopes of the regression of for the Ogle host line and the regression for the Otana host line. Pairwise comparisons of slope are only shown for the Otana host line since this is the only host line that had differed significantly in slope. All estimates and standard errors are from the full ANCOVA model used to analyze sporulation capacity (Table 5).
