# Supplementary material for: Pathogen and host genotype differently affect pathogen fitness through their effects on different life-history stages
Source: BMC Evol Biol. 2012 Aug 2;12:135. doi: 10.1186/1471-2148-12-135 (PMC3483255; doi:10.1186/1471-2148-12-135)
Supplement: Additional file 5 — Summary of ANCOVA results for sporulation capacity for datasets with either of the Pendek or Pendek38 host genotypes excluded. [file 1471-2148-12-135-S5.pdf]

**Additional file 5. Summary of ANCOVA results for sporulation capacity for datasets with either of the Pendek or Pendek38 host genotypes excluded.**

| | Pendek 38 excluded | | | | Pendek excluded | | | |
|---|---|---|---|---|---|---|---|---|
| **Source** | **DF** | **Type II SS** | **F** | | **DF** | **Type II SS** | **F** | |
| Block | 1 | 3.026 | 8.15 | ** | 1 | 3.294 | 9.13 | ** |
| Pathogen | 4 | 3.924 | 2.64 | * | 4 | 3.728 | 2.58 | * |
| Whole plot error | 44 | 16.327 | 14.98 | ** | 42 | 15.156 | 8.70 | ** |
| Pustule density | 1 | 1.456 | 58.77 | *** | 1 | 0.464 | 11.18 | *** |
| Host | 3 | 1.959 | 26.36 | *** | 3 | 1.991 | 15.99 | ** |
| Host * density | 3 | 0.475 | 6.39 | * | 3 | 0.401 | 3.22 | |
| Pathogen * Host | 12 | 0.803 | 2.70 | | 12 | 1.308 | 2.63 | |
| Split plot error | 8 | 0.198 | | | 9 | 0.373 | | |

Log transformed total spore production (mg spores per $cm^2$ of leaf tissue) was used as the response variable and log transformed pustule density (pustules per $cm^2$ of leaf tissue) was used as the covariate. Asterisks indicate significance at $p < 0.05$, 0.01, and 0.001, respectively. Excluding Pendek38 or Pendek does not change the significance of pathogen, host, or interaction effects detected in the full model (Table 6). Excluding Pendek does decrease the significance of the host*density effect (p=0.076).
